# From Delivery to Adoption of Physical Activity Guidelines: Realist Synthesis

**DOI:** 10.3390/ijerph14101193

**Published:** 2017-10-08

**Authors:** Liliana Leone, Caterina Pesce

**Affiliations:** 1 CEVAS Center for Research and Evaluation, 00175 Rome, Italy; leone@cevas.it; 2 Department of Movement, Human and Health Sciences, University of Rome “Foro Italico”, 00135 Rome, Italy

**Keywords:** guidance implementation, multisectoral policy, realist review, knowledge translation, organisational change, health, active living, urban planning

## Abstract

Background: Evidence-based guidelines published by health authorities for the promotion of health-enhancing physical activity (PA), continue to be implemented unsuccessfully and demonstrate a gap between evidence and policies. This review synthesizes evidence on factors influencing delivery, adoption and implementation of PA promotion guidelines within different policy sectors (e.g., health, transport, urban planning, sport, education). Methods: Published literature was initially searched using PubMed, EBSCO, Google Scholar and continued through an iterative snowball technique. The literature review spanned the period 2002–2017. The realist synthesis approach was adopted to review the content of 39 included studies. An initial programme theory with a four-step chain from evidence emersion to implementation of guidelines was tested. Results: The synthesis furthers our understanding of the link between PA guidelines delivery and the actions of professionals responsible for implementation within health services, school departments and municipalities. The main mechanisms identified for guidance implementation were scientific legitimation, enforcement, feasibility, familiarity with concepts and PA habits. Threats emerged to the successful implementation of PA guidelines at national/local jurisdictional levels. Conclusions: The way PA guidelines are developed may influence their adoption by policy-makers and professionals. Useful lessons emerged that may inform synergies between policymaking and professional practices, promoting win-win multisectoral strategies.

## 1. Introduction

Worldwide trends of inactivity, which increase with economic development urge societies to invest in physical activity (PA) promotion to counteract the growing social and economic costs of multiple non-communicable diseases associated with insufficient PA levels [1]. In line with the global action plans on PA of the World Health Organization [2], supranational and national institutions have provided recommendations on promoting health-enhancing PA across sectors and age groups [3]. Whether or not people choose to engage in health-related behaviours as PA depend on a multiplicity of factors. Ecological multilevel models have been applied to PA for explaining the multiple levels of influence on PA behaviours and the interactions across individual, interpersonal, environmental and policy levels of influence [4,5]. Ecological models of health promotion applied to PA clearly show that the multi-level processes that shape health are beyond the interests and expertise of health disciplines alone. The outcomes of an agreed consensus process (concept mapping) performed by a multi-disciplinary panel of European experts to identify and cluster PA determinants, their level of effect and modifiability have highlighted that the cluster of determinants with highest rating of priority for research was that of supportive environments and, within it, the time spent outdoor [6].

Strong scientific evidence supports the relationship between built environment and trends toward physical (in)activity at population level [7,8], despite some gaps in research designs [9]. Therefore, current PA promotion guidelines are often addressed to policy sectors that are considered strategic, as urban planning and transport. A core objective of public health policies is to support and facilitate the development of multisectoral interventions aimed at reversing the sharp decline in PA. Such policies are endorsed by a number of strategic frameworks and official statements of the European Commission [10] and by evidence-based guidance published by the European Union, the World Health Organisation (WHO) and national health authorities [11,12]. 

Two main types of guidelines for PA promotion have been produced. The first type encompasses official position statements or clinical and educational guidance documents underpinned by scientific evidence on the relationship between PA/exercise and health. Such guidelines consist of recommendations for reaching and maintaining health-enhancing levels of PA, targeting different age groups [13,14], from beginners to conditioned individuals [15]. The second type of guideline concerns PA promotion interventions during daily living or in specific settings such as schools, community and workplace [16,17,18]. This type of public health guidelines, targeted at governments, different departments, sport agencies, education sector and local health organisations [11,19,20], offers recommendations on policy actions to promote and support health-enhancing PA such as walking and cycling, at different jurisdictional levels. 

Guidelines are targeted to disseminate updated information and evidence-based recommendations in order to promote a change in behaviour toward a healthy lifestyle and adherence to recommended health-related practices. However, despite widespread circulation and publicity of clinical and educational health-related guidelines, they are often not applied [21,22]. A wide array of personal, guideline-related and external factors can represent barriers to their uptake and implementation [23]. Moreover, guidelines may be intended to serve as a mere “moral suasion” rather than as tools for more coercive policy action demanding a concerted effort at implementation by policymakers and stakeholders. Consequently, there is a substantial gap between evidence and practice, and potential positive health outcomes remain elusive [24]. To fill this gap, innovative strategies to promote the dissemination of guidelines have been suggested, as well as a better understanding of factors influencing the uptake of effective programmes or policies [23,25,26,27]

The formulation of guidance to influence policymaking and professional practices is embedded in the broader process of knowledge translation [28]. According to the WHO [29], “knowledge translation” is a paradigm that assists in closing the gap between scientific evidence and practice. Along the pathway leading from research evidence to knowledge, wisdom, policy and practice, knowledge translation strategies can help capitalise on scientific evidence to inform policy and practice development. However, the knowledge translation process encounters difficulties in the field of evidence-based policymaking, as stated by Greenhalgh and Wieringa [30] “where it seems that knowledge obstinately refuses to be driven unproblematically into practice” (p. 501).

Multiple reasons explain why research findings and evidence-based guidance cannot be transferred in a simple linear way into policy [23,31,32]. The adoption of guidelines is a process of argumentation where decisions are influenced by feasibility, (un)ambiguity of scientific evidence, resource constrains, existence of many legitimate goals, (dis)agreements about relevance of problems and the system of values which exists. Anthropologic studies have demonstrated that the adoption of guidelines involves socially constructed knowledge mediated by a range of informal interactions in fluid communities of practice. In fact, it seems that clinicians rarely access and use explicit evidence from research or other sources directly, but rather rely on collective, internalised guidelines widely influenced by interactions with opinion leaders and other sources of tacit knowledge [33]. 

Concluding, an incomplete evidence base still exists to decide which guideline dissemination and implementation strategies may be effective; to this aim, a theoretical framework should drive the synthesis of evidence to enhance the adherence of different users [34]. There is a range of factors that are considered preconditions for the introduction of PA guidelines in routine primary health care [35]. However, there still is a lack of knowledge about the implementation of PA guidelines [36] for multilevel interventions targeting individuals, social environments, physical environments, and policies. To fill this knowledge gap, it is necessary to examine the “which evidence for whom” issue, especially focusing on how evidence coming from the health sector is used in the decision-making processes within and across different policy sectors. 

The purpose of this realist review was to develop a conceptual framework of facilitators of and barriers to the delivery and adoption PA guidelines within different policy sectors. 

Specifically, we aimed to identify: 

(1) Mechanisms of advocacy through which PA entered onto the agenda setting and ways in which evidence was translated in PA guidelines; 

(2) Factors that may facilitate or hamper PA guidelines adoption within national plans or regulatory tools in different policy sectors and compliance by relevant practitioners. 

These issues were addressed using the realist synthesis approach [37], which is a systematic theory-driven review method based on a critical realism philosophy of science. According to recent claims, realist synthesis may be helpful to knowledge users in public health [38,39], particularly for synthesizing evidence on effective implementation [32]. Different from meta-analytic techniques, which provide a quantified summary of intervention efficacy, the realist synthesis trades quantification for exploration of causal underpinnings of intervention effects under different context conditions [40]. Although the strength of meta-analysis is to provide a precise estimate of intervention effects while quantifying heterogeneity among results and considering potential moderators, this review technique seems not sufficient to understand how an intervention works. The realist synthesis, though not offering any quantifiable summary of intervention efficacy, is particularly suitable to explain the reasons of success or failure of complex social interventions with long implementation chain [41]. It identifies what it is about a programme that generates changes (i.e., the mechanism) and under which circumstances the mechanism is triggered (i.e., the context), thus changing the decision making of subjects, both of beneficiaries affected by intervention and of decision makers and practitioners responsible for the intervention (i.e., the outcome) [38].

## 2. Methods

The process from evidence emersion to the delivery of PA guidelines and to changing of regulatory tools and professional practices in multiple policy sectors was examined; thus conceptualising the guidance implementation process as a complex social intervention involving a complex implementation chain. Indeed, complex intervention programmes are by definition implemented by different stakeholders. Along the implementation chain, resources and opportunities are provided to and exploited by different decision makers. 

Based on this conceptualization, we used the “realist approach” of Ray Pawson [37] to review literature and followed the Realist And Meta-narrative Evidence Syntheses: Evolving Standards (RAMESES) protocol for standard publication [38]. A central process of a realist review is the construction of an initial programme theory that encompasses assumptions, tacit and explicit “theory of change” by policymakers. The process continues by questioning, testing and refining the programme theory and assumptions by using scientific evidence. Testing the integrity of the programme theory permits verification whether the guidance implementation process flows as predicted, leading to the promotion of evidence-based practices.

### 2.1. Literature Scoping and Initial Programme Theory Development

**Figure 1:**
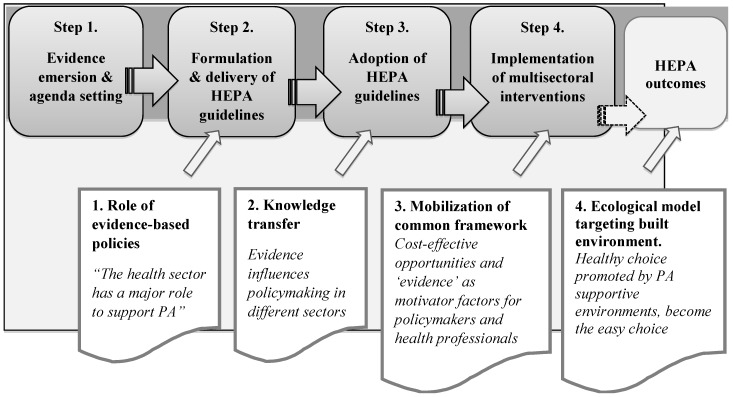
Main steps and assumptions of the initial basic programme theory: the chain from health-enhancing physical activity (HEPA) guidelines delivery to practices.

To map and articulate the key programme theories [37,42] of PA guidelines utilisation, we started out scoping literature and analysing governmental plans, official statements, strategic plans for PA promotion, PA guidelines from databases of main institutions and international networks (European Union—EU, WHO, Public Health Agencies), as well as explicit theory of guidelines dissemination and implementation [43]. The logic of basic programme theory construction adopted in previous realist syntheses concerning public health regulatory tools and legislations [44] guided our simplified initial construction of the programme theory. Particularly to develop the sequential stages of our realist review (Figure 1), we moved from evidence emersion (Step 1) to guidance enforcement and implementation of multisectoral interventions (Step 4). Figure 1 includes these steps and illustrates the underlying assumptions, how evidence-based PA guidelines are meant to work, and what impacts they are expected to have. The four main sequential stages are: emersion of scientific evidence and definition of an agenda setting (Step 1); formulation and dissemination of PA guidelines (Step 2); adoption of PA guidelines within national PA plans and regulatory measures at local level (Step 3); implementation of interventions for PA promotion and changes of professional practices within several policy sectors (Step 4). These four main steps of the initial programme theory are logically linked to each other through some propositions (also named assumptions). 

The general rationale of the initial programme theory is grounded on the evidence-based policy movement and on the key role attributed to guidelines as a tool to influence policymaking processes and, lastly, practitioners’ behaviour [45]. Specifically, the assumptions underlying the progression through the four steps are drawn from core documents highlighting: (1) the leadership role that should be taken by the public health sector for PA promotion [46]; (2) the relevance of mechanisms and theoretical models of “knowledge transfer” and “knowledge translation” for processes of PA guidelines diffusion and utilisation [30,47]; (3) the importance of developing a common agenda for PA [48] to foster common understanding between sectors [49]; (4) the relevance of ecological models embedding the built environment [50].

For each step, possible realist explanations regarding mechanisms that underpin change in specific contexts were inferred from the reviewed literature by answering a number of specific sub-questions. To generate and prioritize the review sub-questions, we conducted a consensus process utilising the content expertise of policy makers and scholars of PA promotion. To this aim, a sheet was sent to four targeted experts from the Health-Enhancing PA (HEPA) Europe network working at national/European level to obtain suggestions about the consistency of the initial programme theory and judgments about the relevance of the related questions. The sheet included a set of questions centred on the promotion of PA for health. HEPA experts’ feedback underlined the need to monitor, at European and national level, the effectiveness of PA guidelines implementation.

The sub-questions for each step were mainly expressed in terms of possible hypotheses to test Context-Mechanism-Outcome configurations. In a realist review, a Context-Mechanism-Outcome pattern configuration allows to retrace regularities and to understand “what works for whom in which circumstances” [37]. For example, advocacy for health, defined as actions targeted to gain political commitment, policy support, social acceptance and systems support for a particular health goal or programme, is well recognized as a priority for health promotion strategies [51]. Thus, we deemed relevant to understand how in the EU Union (Context) and through which type of advocacy (Mechanism) PA entered onto the policy agenda (Outcome) and therefore formulated a corresponding question (question 1.1 in the Results section). 

### 2.2. Searching Process

Primary studies, reviews and evaluation studies were initially searched through PubMed, EBSCO, Google Scholar and, according to the realist synthesis approach, continued through the snowball technique with continuous iterative search. Policy documents were mainly retrieved from European Union, European Commission, WHO and WHO Europe websites. The search strategy involved intersections of the following terms: physical activity; physical inactivity; health-enhancing physical activity; physical exercise; active commuting; guideline; guidance; guidance implementation; guidance dissemination; health promotion guidance; plan; knowledge translation; built environment; evaluation; agenda setting; advocacy; delivery, adoption; implementation; multisectoral intervention(s); multisectoral strategy/-ies; policy; healthy environment; school; education; sport; transport; community; partnership; local authority.

**Figure 2:**
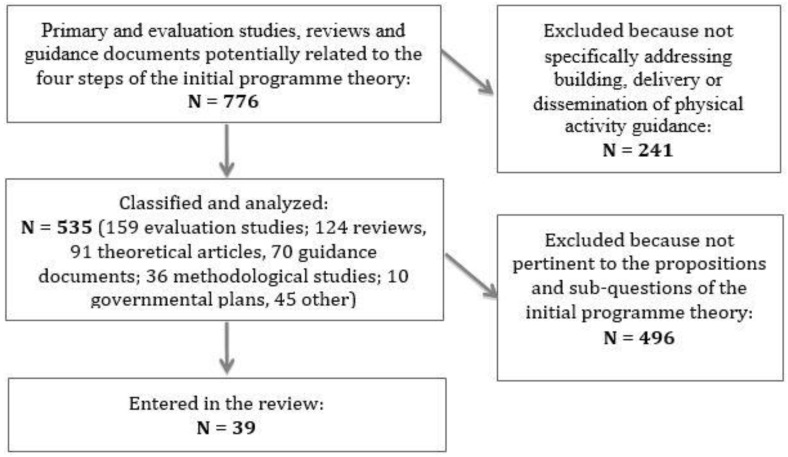
Selection strategy flow diagram.

It is important to underline that for realist synthesis in general and for our review questions in particular, relevant data sources go beyond traditional disciplinary and sector boundaries. Seven hundred and seventy-six electronic references were collected considering all relevant research designs and methods; 535 references covering publication years 2002 to 2017 (date last searched: August 2017) were analysed. A first selection was performed excluding those publications not specifically addressing evidence emersion, building, delivery or dissemination of PA guidance. In a subsequent selection step, references were included according to their pertinence to the four propositions and specific sub-questions. At the end of the process, we selected 39 studies, among them seven systematic reviews, in order to respond to the sub-questions related to each step of the initial programme theory testing. The selection strategy is represented in Figure 2. A supplemental file shows the entire initial set of references (File S1: Dataset_PA_review.xls).

### 2.3. Selection and Appraisal of Documents

The inclusion of data to inform programme theory development was guided by the RAMESES principles of relevance and rigour [38]. The quality assurance of primary sources, as requested in a realist synthesis, concerned the process of theory testing and the inferences that were appraised, rather than the judgment of each contribution. The selection process was iterative and driven by the need to identify data for developing, refining or testing the programme theory, going beyond a hierarchical prioritization of evidence based on quality assessment parameters as study design (e.g., privileging randomised controlled trials). Each selected study contributed to answering the review questions by testing the theoretical assumptions, questioning and refining the initial programme theory. 

The typical Context-Mechanism-Outcome realist configuration was adopted to compare and interrogate literature and synthesise evidence. According to the scope of this review, we attempted to identify recurrent patterns of contexts and outcomes, giving priority to the following contexts: (1) main policy sectors (urban planning, transport, health, education, sport), (2) different governmental levels (supranational, State, local authority, scholastic district), (3) country areas with a special focus on the EU context. Successively, we sought to explain these recurrent patterns or demi-regularities through the mechanisms by which they occurred. All the assumptions, corresponding to each of the four steps of the initial programme theory, were tested through comparison with literature search results. 

## 3. Results

### 3.1. Step 1: Emersion of Scientific Evidence and Definition of an Agenda Setting

#### 3.1.1. Sub-Question

- How and Through which Mechanism of Advocacy did PA enter onto the Agenda Setting?

For this first step concerning how clinical and epidemiological evidence influences policymaking processes and how it mobilises interest groups for PA, we chose to focus on the case of EU compared with the United States and Australia. The focus on Europe, contrasted with other main developed economies with analogous inactivity epidemics, was prompted by the actual interest of the European Union institutions for multisectoral PA promotion strategies [3,10,11]. To influence policymaking processes so that PA enters onto the Agenda setting, guidelines must be paralleled by advocacy strategies, that are a combination of individual and social actions designed to gain policy support and social acceptance [48,52]. In the past, in the EU—compared to United States or Australia—there has been a frail advocacy for actions to counteract obesity and increase PA levels [53,54]. Suggested reasons were national governments jealousy of health policy sovereignty, lack of a powerful umbrella of non-governmental organizations and delay of civil society and public health to enter in a mutual moral obligation to prioritise the fight against obesity.

Our findings indicate that the current EU attention to obesity and physically (in)activity in strategic documents [10] depends on the confluence of multiple mechanisms and related outcomes (Table 1, Step I). (1) A mechanism of scientific legitimation underlies the dissemination of the anti-obesity discourse prompted by the WHO scientific view. (2) A mechanism of advocacy leads to the introduction of the EU into a novel policy area pushed by supranational institutions, which attract relevant interest groups toward nutrition-related policies. (3) A cascade effect (“political spillover”) from nutrition policies drives PA into the agenda of EU institutions [53,55]. Nevertheless, there have been unintended effects of EU policymaking, due to the fact diet and obesity issues have been linked to agricultural policy interests. Insofar the role of PA became secondary, as driven by nutrition policy and negatively framed in terms of “battle” against obesity. 

The risk inherent in linking PA promotion mainly to obesity prevention and reduction is that to neglect evidence on the far broader role played by PA in health enhancement and maintenance independently of weight loss and, vice versa, by physical inactivity in the development of major non-communicable diseases [56]. Furthermore, emerging evidence that scales down the overemphasized role of PA in weight control, as in the case of the study of Metcalf and colleagues [57], may lead to an even deeper under-consideration of the pivotal role of PA in holistic health promotion. This subordination of PA promotion to the fight against obesity is reflected in the recent EU mainstream of research within the Joint Programme Initiative “A healthy diet for a healthy life” [58] that under-considers policy sectors relevant for active lifestyle such as transport, urban planning and education. 

### 3.2. Step 2: Formulation and Dissemination of PA Guidelines

#### 3.2.1. Sub-Questions

- Are PA guidelines developed synthesising data meaningful for different policy sectors?
- Is there coherence among PA guidelines delivered by different authorities?

#### 3.2.2. The Source of Evidence

Guidance delivered by public health authorities having scientific legitimation, plays a relevant role in orienting policymaking processes toward PA promotion [2]. Nevertheless, bias in evidence gathering may emerge in PA guidelines due to a lack of consultation and a self-referential search process limited to medical disciplines. In health databases, the distribution of evidence seems skewed by the criterion of “gold-standard” and “hierarchy” of evidence that favours individual level randomized controlled trials (RCT) [59]. This leads to a large neglect of non-medical evidence on intervention categories for promoting active commuting that involve urban transport policies, as health walks and parking charges. The mechanism of scientific legitimation triggered by dissemination of evidence-based guidelines by health authorities may be hindered by a self-referential concept of knowledge and narrowly defined methodological standards that undermine the value of PA guidelines particularly for policymakers of the urban planning and transport sectors (Table 1, Step II). Analysing aspects of the built environment relevant for public health with a cross-disciplinary approach highlights only a minimal overlap between medical and built environment or social science databases [60]. Furthermore, appropriate consideration should be given to grey literature, unpublished reports or institutional documents from the transport sector that inform about targeted behaviour change programmes.

#### 3.2.3. Proliferation of Guidance and Contrasting Evidence

Differences in PA guidelines among regional health departments of the same country sometimes emerged as regards purposes, evidence, focus of prescriptions and optimal amount of exercise [61]. In 2006, the National Institute for Health and Clinical Excellence [62] stated that the evidence base of guidelines was not sufficiently consistent to recommend the use of exercise referral schemes to promote PA. Foster [63] discussed how in the meantime, a further guidance, a policy paper of the UK Department of Health recommended implementing “different” referral schemes for successful prescription. Policy decisions seem to have evolved as parallel lines, with the political community being in conflict with the practice community on this matter due to a lack of consultation at the start of referral question setting [63,64].

### 3.3. Step 3: Adoption of PA Guidelines within National PA Plans and Regulatory Measures at Local Level

#### 3.3.1. Sub-Questions

- Are PA guidelines coherently integrated within national/regional plans for PA, physical education at school and sports promotion?
- Which are the determinants of effective PA guidelines adoption in the health sector at local government level?
- Is the perception of PA as a policy priority influenced by policy sectors different from health (transport, urban planning)?
- Did medical schools adopt curricula coherently with PA guidelines?

#### 3.3.2. Adoption of PA Guidelines within the Education and Sport Sectors

The WHO Europe [65] highlighted the weak level of political commitments and non-synergic relations of PA policies among health, education and sport sectors in plans and documents concerning PA promotion and sport strategies in the European Union (EU) [66]. In the education sector, less emphasis is placed on Physical Education in view of increasing academic demands and economic constraints. Although there still is a limited awareness of the evidence base for the development of quality Physical Education legislation, the importance attributed to Physical Education is increasing also in the United States [67]. Policy mandates for PE in schools seem relevant to ensure PA adherence at school district-level [68]. While in the United States, enforcement state regulations for childcare seem important to a policy approach to physically active childcare settings (Table 1, Stage III), there seems to be a high variability among state regulations and a low compliance with national health and safety guidelines [69]. In the United States, the progression of policies on Physical Education between state and district level varies across states as a function of the strength of the mechanism of health advocacy at state level. It shows a mixture of top-down and bottom-up models, with states leading or reacting to district policy changes [70]. 

In the sport sector, elite sport facilities and events are often prioritised at the expense of those for recreational sport-for-all based on the erroneous assumption that such prioritisation would increase general participation in sport. Indeed in Europe, there is a paucity of evidence supporting the hypothesis that “Sport for All” and elite sport benefit each other [71] and that hosting Olympic Games may lead to increased PA and sport participation for host countries [72]. In contrast, elite sports may deter individual participation because of a perceived competence gap [73]. Therefore, the World Health Organization [65] called for more consideration, in urban planning, of economic investment in recreational sport facilities for the general public, avoiding a prioritisation of elite sport investments. In sum, while the existence of strategies and plans at national level may be an appropriate context for PA guidelines to be adopted within national sport plans, on the other side the same mechanism of enforcement seems to underlie a trade-off between investments for elite sport and sport-for-all at the expense of the latter (Table 1, Step III).

#### 3.3.3. Adoption of PA Guidelines in the Health Sector and Differences among Jurisdictional Levels

State funding for PA, adequate staffing and supportive state legislature seem relevant to the adoption of PA guidelines in the health sector [65]. A mechanism of enforcement of PA guidelines is necessary to their translation into ordinary policy that, in turn, results in increased adoption and implementation. However, the degree of influence of the mechanism of enforcement on the adoption and implementation of PA guidelines seems to depend on the jurisdictional level of public bodies and practitioners (State, local authority, scholastic district) (Table 1, Step III). Among the factors that have been found to support or inhibit the uptake of effective programmes and policies is the degree of authority to implement interventions. 

Guidelines dissemination seems to translate into increased adoption and implementation by state level practitioners more clearly and consistently than by local level practitioners. In The Netherlands, local government is required by national law to develop health policy at the municipal level. Nevertheless, local health policy did not appear to be of pivotal importance to the operations or organization of stakeholders mainly due to the dominance of a medical approach to health that slows the progress of integrated health policies [74]. In Norway, a “Health in all Policies” principle, implying that health inequities may be counteracted when policies and actions are implemented in all sectors, underlies public health policies. However, interventions to improve diet and PA are still not consistently embedded into a multisectoral framework and the sectorised governmental organization hampers the actors at local governance level primarily involved in the implementation [75]. The determinants of policy action for the adoption of PA guidelines also differ as a function of the jurisdictional level. In the EU, the perception of opportunities seems higher at national than local level, whereas the perception of obligations for PA promotion seems stronger by regional than national policy makers [76].

#### 3.3.4. Adoption of PA Guidelines in the Transport and Urban Planning Sectors

In the United States, urban planners seem to play a more relevant role in PA promotion as compared to local health departments, being able to anticipate innovative, multisectoral win-win strategies for active lifestyle and commuting [77]. A public health rationale for urban planning has the potentiality to gain attention and add credibility [36], particularly if PA promotion goals are framed in terms of other dominant concerns for urban planners as liveability, climate change, dynamic centres or socioeconomic development. In Australia, strategies to create PA supportive environments at municipal level have been adopted within the current regulatory framework. Interventions aimed at improving environments for walking, cycling and active recreation received general public support, though there were also barriers to change due to limited funding to realise new ideas and competing priorities [78]. The two main factors that diminished the perceived usefulness of evidence-based guidance by planning and transport professionals were that (1) they were inundated with other guidance and legislation and (2) the guidelines replicated approaches already in existence [36]. More emphasis should be placed, within PA guidelines, on evidence that environmental approaches aimed at changing the structure of physical and organizational environments demonstrate greater success when compared to informational and social-behavioural approaches [79]. 

#### 3.3.5. Medical School Curricula and PA Guidelines Contents

Finally, the so-called “Cinderella” status of PA in health care and public health [80] seems related to the widespread omission of basic teaching elements in the medical school curricula. As demonstrated by Weiler and colleagues [81] in the UK, only a small minority of medical schools included PA in each year of the undergraduate courses, with a negligible amount of time spent on teaching PA compared to pharmacology. There is a delayed translation between PA guidelines and the formal contents of official curricula for medical doctors and the mechanism of advocacy for PA is too weak (Table 1, Step III). This knowledge gap, as discussed below, causes a lack of specific skills and knowledge that, in turn, may act as a mechanism that negatively influences doctors’ attitudes toward exercise prescription and referral schemes.

### 3.4. Step 4: Implementation of Interventions for PA Promotion and Compliance by Actors in Different Sectors

#### 3.4.1. Sub-Questions

- Do school principals’ beliefs about compatibility of PA and academic achievement matter?
- Do medical doctors’ attitudes influence PA prescription?
- Do urban planners’ and administrators’ attitudes influence PA promotion interventions?

#### 3.4.2. School Principals’ Beliefs about Compatibility of PA and Academic Achievement Goals 

School principals who have a supportive attitude to students walking to school seem more likely to believe that active travel benefits not only the health of students, but also their scholastic success [82]. Thus, a mechanism that may render PA goals meaningful is the belief that the time devoted to PA is not detrimental, but even advantageous for academic achievements [83,84]. This has been recently supported also by neuroscientific evidence [85]. The growing body of literature on this issue has recently led one of the most authoritative scientific organizations in exercise and sports sciences (American College of Sports Medicine) to publish the first position stand on this issue [86]. In summary, enhancing PA at school by increasing curricular physical education hours, adding classroom-based physical activities, or exploiting recess time for PA benefits school achievement, or is at least not detrimental to it. This evidence base has important implications for public health and education in school age children [87]. This evidence base can facilitate the upgrade of physical education from the ‘Cinderella’ status to a central role in holistic education. Nevertheless as stated in Step I, in policy documentation and academic literature there is a prevalent discourse on obesity epidemics that narrowly frames PA as mere means of energy expenditure to combat obesity [88]. The dissemination of conceptual models better representing the holistic contribution of PA to human development [89] may be supportive of positive attitudes of school leaders toward enhanced PA. 

#### 3.4.3. Attitude toward PA Prescription by General Practitioners and Clinicians

Lack of time to promote PA is the most often cited perceived barrier [90,91,92,93]. The adoption of clinical behaviours adherent to PA guidelines is influenced by the individual characteristics and the active lifestyle of health care professionals (e.g., high PA levels and self-efficacy, healthy weight status and lifestyle) more than by their organisational systems or intention to prescribe PA. Social-cognitive models used to predict and promote healthy behavioural changes in patients seem unable to predict physicians’ clinical behaviours adherent to guidelines [94]. Instead the mechanism of being a “faithful credible model” for patients seems to be a better explanatory mechanism of why normal weight and physically active health professionals had more confidence in weight management practices [92] (Table 1, Step IV). 

#### 3.4.4. Attitude toward PA Promotion Interventions by Urban Planners and Administrators

The perception of “feasibility” by public officers and administrators of multisectoral policy guidelines seems a relevant mechanism to increase the potential of successful policy implementation to promote an environment more conducive to PA among children [95]. Further related mechanisms emerged that could explain the observed results. A higher familiarity with the concept of active-friendly environments seems related to the perception of a higher feasibility of policy measures, which, if already applied in some neighbourhood, could generate positive contamination and be applied elsewhere (Table 1, Step IV). 

## 4. Discussion

Evidence-based PA guidelines delivered by international health bodies play a significant role for the dissemination of PA recommendations and the realization of multisectoral strategies for PA promotion. However, there is not a simple direct relationship between scientific evidence, delivery of guidance, adoption of policy measures to contrast physical inactivity trends and changes in professional practices. 

The general purpose of the present realist review was to develop a conceptual framework of facilitators of and barriers to the delivery and adoption PA guidelines within different policy sectors. We analysed the long chain leading from advocacy to adoption of PA guidelines and, specifically, (1) the mechanisms of advocacy and the ways of evidence translation into PA guidelines; (2) the factors that may facilitate or hamper PA guidelines adoption in different policy sectors and the compliance by relevant practitioners. Expected and unexpected outcomes are explained by a plurality of mechanisms triggered at each step of this complex implementation chain (Table 1).

Several barriers hamper PA guidelines delivery and adoption. They range from factors that limit the meaningfulness of evidence and the subsequent translation into coherent and useful recommendations for different target groups (e.g., general practitioners, urban planners) to weak mechanisms of advocacy for PA promotion. The main weaknesses identified regarding the delivery and adoption of PA guidelines were (Table 2): (a) PA subservience to the obesity and nutrition issues in the policy agenda; (b) data gathering limited to health databases, conflicting advice among guidelines and lack of consultation; (c) non-synergic relationship, within national PA plans regarding the goals of the sport, education and urban planning sectors; (d) underestimation of the role played by personal beliefs, perceptions and attitudes of public officials and health practitioners. 

Table 2 graphically represents the main results: the arrows signal the threats to the integrity of the original four step programme theory. The differences of institutional contexts explain why PA entered onto the EU policy agenda in a delayed fashion when compared with the United States, Canada and Australia. In addition the PA agenda development was mainly driven by the obesity epidemic challenges and food policy. With few exceptions, the integration of a new area of policymaking on obesity and PA in the EU was mainly driven by a top-down, evidence-based directive by the WHO and the European Commission General Directorate for Health, rather than by a bottom-up pressure by member states governments, non-governmental organizations, public health agencies and professional associations. 

A critical point of evidence-based guidelines is that a medical approach dominates “how” evidence is built and data gathering is fulfilled. It implies some limitations in research agendas about PA, involvement of relevant sectors of knowledge, evidence search techniques, evaluation methods and source of evidence narrowed to health-related databases. According to some scholars, broad conceptual and methodological gaps need to be addressed to progress research on public open space and PA and therefore to better design PA guidelines [96]. When inconsistent evidence emerged among PA guidelines, the main gaps depended on the lack of consultation processes among main stakeholders particularly in the phase of definition of review questions.

Threats to the adoption of PA guidelines within national or local strategic plans and regulatory measures emerged. A key problem is that while the health sector, mainly at national or international level, usually provides leadership or stewardship for PA and publishes guidelines; it then also needs to engage other sectors in the quest for promoting PA. Indeed, the action on PA often falls in the domain of professionals belonging to other sectors such as education, sport urban planning and transport [46]. Non-synergic relations of national plans of health-enhancing PA policies by EU member states with goals of education, sport and urban planning policies are due to competition for resources, different perceptions of need for and feasibility of PA promotion, and absence of a common understanding and agreement about the expected outcomes. 

PA guidelines seem more effective for urban planning and transport sectors if framed in broader terms (i.e., not only physical health promotion) and inspired by a holistic view of health that encompasses other dominant concerns as quality of life and environmental sustainability. Effective PA promotion strategies can be implemented by coordinators of the built environment and assisted by academics with research expertise in architecture, planning and health [97]. Effective partnerships are based on win-win strategies, namely the linkages between active lifestyle, active commuting, traffic reduction, pollution and dependence from fuel transportation [98]. Urban planners often play the role of “insider innovators”, reducing the barriers among technical staff and anticipating multisectoral strategies. In contrast, local health services seem not to play a pivotal role in evidence-based PA promotion by means of community plans, or mixed land use [77]. 

Results demonstrated that the adoption of, or adherence to PA guidelines are influenced by factors acting at different community and individual levels: previous experiences of effective PA promotion interventions in the community, competition among different priorities or other health promotion guidelines [64], as well as personal characteristics, lifestyle and attitudes towards PA guidelines of policy-makers and clinicians. Factors impacting on adequate counselling and prescription by physicians are their lack of knowledge of PA and the potential conflict with their personal lifestyle. Thus, expanding physicians’ academic curricula to include PA in their professional identity building [99] and devoting more attention to their motivations, lifestyle and cognitive distortions is warranted to better tailor knowledge translation and PA guidelines dissemination.

Many mechanisms mediate the long journey that goes from evidence emersion to PA promotion guidelines delivery and adoption by several sectors. Some of the mechanisms were not present in the initial programme theory, but emerged along the review. Though the role of scientific knowledge is widely recognised, the adoption of PA guidance varies and the goals are reframed according to each policy sector, its priorities and dominant organisational culture. For example, school principals and their beliefs about the compatibility of physical education with academic performance represents a key issue, while for urban planners at municipal level, active commuting and cycling are stronger priorities than PA “per se”. In the sport sector, PA policy competes with policies that prioritise investments for elite sport facilities and events. In conclusion, policy sectors other than health seem to play a pivotal role for ensuring effectiveness of health-enhancing PA promotion. Thus, it seems necessary to extract evidence and offer recommendations that are meaningful for other sectors.

To our knowledge, this review represents the first attempt to employ the realist synthesis approach to analyse the delivery and adoption of PA promotion guidelines more broadly in several policy sectors. It may complement review outcomes focused on the uptake of clinical guidelines in the health sector obtained with a realist synthesis approach [22].

However, in each policy sector, the concepts of “evidence” and “evidence-based policy” differ in terms of quality assessment and the relevance of evidence in specific circumstances [100]. The present preliminary review did not analyse the implementation of PA guidance in depth in each policy sector. Giving the novelty of applying the realist review approach to the implementation of PA promotion guidelines, the initial programme theory with its main assumptions (Figure 1), tested and refined by means of the present realist synthesis (Table 2) could be further optimised by future studies focusing on specific policy sector contexts.

## 5. Conclusions

In the present review, the realist synthesis approach was used to investigate variability in the adoption of PA guidelines in different circumstances, namely at different levels of governance and in different policy sectors [39,101,102]. In fact, it helped further our understanding of how to promote effective strategies for knowledge translation and guidelines dissemination, narrowing the gap from evidence to evidence-based policy. 

As evidence and guidance delivery alone are not sufficient to influence practitioners and policy maker behaviours [34,36,75], the review analysed key steps of the long path that goes from the agenda setting to guidelines adoption and implementation, highlighting specific threats at each step. The process of agenda setting within the policy arena, with mechanisms of scientific legitimation and advocacy, was found to influence the adoption of PA priorities at supranational and national level, with nutritional policies driving the introduction of PA into EU agenda. Although PA guidelines delivered by health authorities, because of their scientific legitimation, play a relevant role in all the analysed sectors, different mechanisms are involved in the education, sport, transport and urban planning, sectors. Within the transport and urban planning sectors, dissemination is influenced by knowledge translation mechanisms, with PA goals and concepts reframed in terms of policy strategies addressing sustainability. Other mechanisms influencing PA guidelines implementation are positive expectations of school principals, health practitioners’ PA behaviours and perception of being a faithful role model, and perception of feasibility by urban planners. This broad range of identified causal mechanisms should be considered in both policymaking and practice.

Our conclusion is that PA guidance delivery should go beyond a narrow focus on the “know-do gap” [30], acknowledging the relevance of evidence that comes from sectors where RCTs, that are object of traditional forms of systematic review, are not common (e.g., urban planning or transport). Furthermore, greater efforts are needed to monitor the implementation of guidelines at different jurisdictional levels, as relevant policies for PA promotion are not made by national Governments, but at the regional and local level [31]. For effective guidance delivery, particular attention should be devoted to factors that go from consultation with researchers and policy makers of different policy sectors to organisational incentives for promoting active living strategies at local level and positive personal attitudes and behaviours of practitioners at individual level. 

## Figures and Tables

**Table 1:** Examples of context-mechanism-outcome configurations.

Step	Context	Mechanism	Outcome
I° Agenda setting and advocacy	Supranational health body advocating for PA within an “obesity fight” framework	Scientific legitimation	+ Shift of relevant PA interest groups to supranational level
	Lacking pressure by other public bodies and NGOs	Advocacy	− PA introduction into EU agenda driven by nutrition policy.
		“Cascade” effect	
II° PA guidelines delivery	Health sector delivering PA guidelines addressed to several policy sectors	Scientific legitimation	+ Role of public health authorities as credible sources
		Self-referential concept of knowledge	− Bias in evidence gathering (solely from health databases) and lack of consultation with other policy sectors
III° PA guidelines adoption	Scholastic districts with strong state policies for child health care	Advocacy	+ Adoption of PA guidelines by the education sector
		Enforcement	+ Enhanced PE at school
	Sport strategies and plans at national level	Lacking advocacy	+ Partial adoption of PA guidelines within national sport plans
		Competition for attracting resources	− Investments for elite sport facilities at the expense of PA/sport for all
	Health authorities at state level	Enforcement due to higher authority	+ Dissemination of PA guidelines resulting in increased adoption and implementation at state level
	Health authorities at local level	Lacking enforcement	− No pivotal role due to the dominance of a medical approach to health
	Transport and urban planning sectors at local government level	Knowledge translation	+ Reframing of PA goals within each policy sector
			+ Promotion of urban environments supportive for PA with a pivotal role of urban planners as innovators
		Enforcement	− Perception of recommendations as “statements of the obvious” without link to legislation.
	Medical schools	Knowledge gap	− Lacking or limited introduction of PA subject in medical curricula
		Lacking advocacy	
IV° Implementation	School principals’ believes about compatibility of PA and academic achievement.	Enforcement	+ Support for PA in in-school and out-of-school settings
		Positive expectations	
	General practitioners’ habits to practice PA	Perception of being a faithful model	+ High adherence to PA guidelines, frequent PA prescription to patients
		Lacking PA skills and habits	− Low adherence to PA guidelines, seldom PA prescription to patients
	Urban planners’ and administrators’ attitudes towards PA promotion interventions	Perception of feasibility	+ Implementation of policy measures for active-friendly environments for children
		Familiarity with concepts	

Note: “+” sign means positive outcome, while “−“ sign means negative outcome.

**Table 2:** Realist synthesis findings: threats, confirmation and refinements of the programme theory.

Confirmation of the Initial Programme Theory	Refinements or Threats of the Initial Programme Theory
**STEP 1: Agenda Setting** 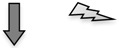	PA introduction into the EU policy agenda with a strong delay and driven by obesity epidemic.
**STEP 2: Guidelines Delivery** 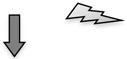	Gaps due to gathering data limited to health databases. Methodological concerns and lack of consultation. Possible conflicting advice in some PA guidelines.
**STEP 3: Guidelines Adoption** 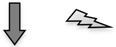	Non-synergic relations in EU PA plans among health, education and sport sectors. Prioritization of elite sport facilities. Weak role played by local health agencies. Risk of absence of added value in PA guidelines for urban planners.
**STEP 4: Evidence-based Implementation** 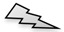	Role of personal lifestyle and believes on PA counselling and prescription by health care professionals. Role of active-friendly environments concepts among administrators and urban planners.

## Supplementary Materials

The following is available online at www.mdpi.com/1660-4601/14/10/1193/s1, File S1: Dataset_PA_review, listing the complete set of literature consulted (until August 2017).
